# Hypoxia Promotes Osteoclast Differentiation by Weakening USP18-Mediated Suppression on the NF-κB Signaling Pathway

**DOI:** 10.3390/ijms26010010

**Published:** 2024-12-24

**Authors:** Xiaoxia Fan, Botong Li, Shengjun Chai, Rong Zhang, Chunmei Cai, Rili Ge

**Affiliations:** 1 Research Center for High Altitude Medicine, Qinghai University, Xining 810001, China; myselfxiaofan@163.com (X.F.); ibt6096@163.com (B.L.); ys221002100819@qhu.edu.cn (S.C.); 19277065912@163.com (R.Z.); 2 Key Laboratory of the Ministry of High Altitude Medicine, Qinghai University, Xining 810001, China; 3 Key Laboratory of Applied Fundamentals of High Altitude Medicine, Qinghai-Utah Joint Key Laboratory of Plateau Medicine, Qinghai University, Xining 810001, China; 4 Laboratory for High Altitude Medicine of Qinghai Province, Qinghai University, Xining 810001, China

**Keywords:** osteoporosis, osteoclasts, hypoxia, USP18, NF-κB signaling pathway

## Abstract

Osteoporosis, a prevalent metabolic bone disorder, is characterized by reduced bone density and increased fracture risk. The pathogenesis of osteoporosis is closely associated with an imbalance in bone remodeling, in which the resorption function of osteoclasts exceeds the formation function of osteoblasts. Hypoxia has been implicated in the promotion of osteoclast differentiation and the subsequent development of osteoporosis. The ubiquitin–proteasome system (UPS) and its regulatory enzymes, deubiquitinating enzymes (DUBs), play a significant role in bone homeostasis. In this study, we investigated the contribution and mechanism of Ubiquitin-specific protease 18 (USP18), a DUB, in osteoclast differentiation under hypoxic conditions. BMDMs and RAW264.7 cells were treated with RANKL to induce osteoclastogenesis and were subjected to overexpression or knockdown of USP18 under normoxic or hypoxia conditions. Osteoclast formation was assessed using TRAP staining, and the expression of osteoclast marker genes was determined using qRT-PCR. The activation of the NF-κB signaling pathway was evaluated using immunoblotting. We found that hypoxia significantly enhanced the differentiation of BMDMs and RAW264.7 cells into osteoclasts, accompanied by a notable downregulation of USP18 expression. The overexpression of USP18 inhibited RANKL-induced osteoclast differentiation, while the knockdown of USP18 promoted that process, unveiling the inhibitory effect of USP18 in osteoclastogenesis. Furthermore, the overexpression of USP18 rescued the hypoxia-induced increase in osteoclast differentiation. Mechanistic insights revealed that USP18 inhibits osteoclastogenesis by suppressing the NF-κB signaling pathway, with a potential target on TAK1 or its upstream molecules. This study indicates that hypoxia promotes osteoclast differentiation through the downregulation of USP18, which, in turn, relieves the suppression of the activation of the NF-κB signaling pathway. The USP18 emerges as a potential therapeutic target for osteoporosis treatment, highlighting the importance of the hypoxia–DUB axis in the pathogenesis of the disease.

## 1. Introduction

Osteoporosis, a subclinical condition, is the most common metabolic bone disease globally, characterized by an increased risk of fractures [1,2]. Osteoporosis is fundamentally attributed to an imbalance in bone remodeling, resulting from the bone resorption function of osteoclasts exceeding the bone formation function of osteoblasts [3,4,5]. There are over 8.9 million osteoporosis-related fractures worldwide [6]. Over 33% of women and 10% of men over 50 years will experience at least one osteoporosis-related fracture in their lifetime [7]. With the aging of the population, osteoporosis has become a public chronic disease, with associated fractures, high disability rates, and mortality rates, thereby increasing social and economic burdens [8]. Moreover, an estimated 80% to 90% of adults with osteoporosis do not receive adequate treatment even during the secondary prevention phase, indicating a notable deficiency in the primary prevention of osteoporosis [9]. Therefore, elucidating the novel pathogenic mechanisms of osteoporosis may provide a promising therapeutic approach for clinical treatment.

Oxygen is an essential factor for bone growth, development, and metabolism. Hypoxia, which is defined as the threshold at which oxygen concentration limits normal cell function [10], results from inadequate oxygen supply or excessive consumption, hindering normal cellular metabolism [11]. The oxygen tension in most normal tissues ranges from 2% to 9% (14–65 mmHg) [12], while it is <6.6–8.6% in bone [13]. Hypoxia conditions, such as reduced oxygen partial pressure, poor oxygen diffusion, and perfusion environment, may trigger cellular hypoxic responses. Studies have demonstrated that both osteoblasts and osteoclasts are oxygen-sensing cells [14]. Pathological or environmental hypoxia can disrupt bone homeostasis, thereby affecting bone health [15,16]. Mice exposed to a simulated altitude of 5500 m for 28 days exhibited significant reductions in bone mineral density of the fourth lumbar vertebra, bone volume fraction, trabecular thickness, and trabecular number, indicating that hypoxia may increase the risk of fractures [17]. However, the mechanisms by which hypoxia is involved in osteoclast differentiation require further clarification.

Research has shown that hypoxia can regulate the transcription of numerous deubiquitinating enzymes (DUBs) through the hypoxia-inducible factor (HIF) signaling pathway, thereby mediating the occurrence and development of various diseases [18,19,20]. The human genome encodes approximately 100 DUBs, which are further classified into six subfamilies [21,22,23,24]. In recent years, an increasing amount of evidence has indicated that ubiquitination plays a significant regulatory role in maintaining bone homeostasis [25]. Several DUBs, including CYLD (NF-κB and RANKL signaling) [26,27], USP15 (IκBα) [28], USP18 (Type I IFN) [29], USP26 (IκBα) [30], USP34 (NF-κB signaling) [31], A20 (NF-κB signaling) [32,33], MYSM1 (unknown mechanism) [34], and POH1 (Mitf’s transcriptional activity) [35], have been implicated in modulating osteoclast function. It should be noted that pioneering studies have rarely focused on whether and how hypoxia regulates the expression of DUBs to modulate osteoporosis progression. Additionally, mounting studies gradually revealed that targeting the ubiquitin–proteasome system (UPS) might be a promising strategy for clinical applications of osteoporosis [36,37,38,39,40,41]. Therefore, a thorough understanding of the hypoxia–DUB axis in osteoporosis could not only improve the therapeutic efficacy but also minimize side effects for the specificity of the targeted treatment.

In this study, we found that hypoxia significantly promoted osteoclast differentiation, and USP18 was significantly reduced during RANKL-induced osteoclast differentiation in RAW264.7 cells. Further functional results revealed that USP18 presented as a negative regulator in RANKL-induced osteoclast differentiation. In addition, overexpressing USP18 remarkably rescued hypoxia-enhanced osteoclast differentiation. Mechanically, the USP18-mediated suppression of the NF-κB signaling pathway was responsible for its inhibition in osteoclastogenesis. Thus, hypoxia might weaken the USP18-mediated suppression of the NF-κB signaling pathway to significantly promote osteoclast differentiation, consequently participating in osteoporosis development. Our research provided novel insights into the molecular basis of osteoporosis pathogenesis for developing potential therapeutic strategies.

## 2. Results

### 2.1. Hypoxia Significantly Enhances the Osteoclasts’ Differentiation of Both RAW264.7 and BMDM Cells

To identify optimal osteoclast induction conditions, RAW264.7 cells were cultured in either complete α-MEM or DMEM medium, with the addition of 75 ng/mL RANKL (Figure S1A–C). RAW264.7 cells, which are known for their smaller size and rapid growth, showed noticeable proliferation and clustering after one day of induction. On Day 1, a few scattered TRAP-positive multinucleated giant cells with irregular cell shapes and varying numbers of nuclei were observed in the α-MEM medium. (Figure S1A). By Day 2, the emergence of TRAP-positive monocytes became more apparent, with a growing distinction between the α-MEM and DMEM groups (Figure S1A–C). Notably, on Day 3, a significant increase in TRAP-positive cells was observed (Figure S1A). Some of these giant cells contained dozens of nuclei, clustered either centrally or peripherally within the cells. The cytoplasm of these cells appeared hollow. Remarkably, compared to DMEM, the number of osteoclasts in the α-MEM group was dramatically higher, and the difference was pronounced on Day 3, with a *p*-value < 0.0001 (Figure S1A–C). On Day 4, a large number of mature osteoclasts disintegrated, leading to decrease in the number of osteoclasts (Figure S1A). In conclusion, the α-MEM medium was found to be more effective than DMEM in promoting the RANKL-induced differentiation of RAW264.7 cells in osteoclasts. Therefore, the subsequent experiments utilized the α-MEM medium as the preferred culture condition.

**Figure 1:**
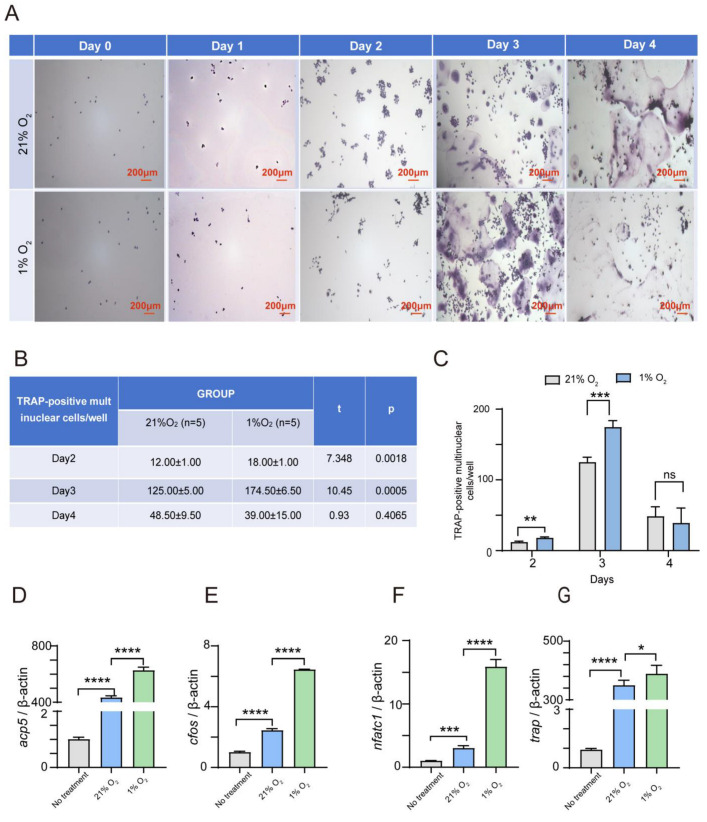
Hypoxia significantly enhances the osteoclast differentiation of RAW264.7. (**A**–**C**) RAW264.7 cells were treated with 75 ng/mL RANKL for 0–4 days under normoxic (21% O_2_) or hypoxic (1% O_2_) conditions. Osteoclastogenesis was measured using tartrate-resistant acid phosphatase (TRAP) staining, and TRAP-positive multinucleated cells were considered mature osteoclasts. Scale bar, 200 μm. *n* = 5. (**D**–**G**) qRT-PCR detected the mRNA expression of osteoclast markers using RNA isolated from RAW264.7 cells cultured with or without 75 ng/mL RANKL for 2 days under normoxic (21% O_2_) or hypoxic (1% O_2_) conditions. The relative mRNA level of individual genes was expressed as the fold induction compared with no treatment (*n* = 3). Data are presented as mean ± SD. (* *p* < 0.05, ** *p* < 0.01, *** *p* < 0.001, **** *p* < 0.0001, ns: not significant).

Importantly, under sustained 1% O_2_ treatment, the number of TRAP-positive multinuclear cells was greater than the normoxic group, particularly from Day 2 to Day 3 (Figure 1A–C). Additionally, sustained hypoxia treatment led to a robust increase in the expression levels of osteoclast marker genes, Acp5, cFos, Nfatc1, and Trap (Figure 1D–G). These findings indicated that sustained hypoxia significantly enhanced the differentiation of RAW264.7 cells in osteoclasts. We further examined the morphological changes in osteoclast formation under short-term hypoxia treatment (24 h exposure to 1% O_2_). Consistent with the effects of sustained hypoxia, there was a notable elevation in the number of TRAP-positive multinuclear cells in the short-term hypoxia group, especially on Day 3, with the most pronounced increase and difference, compared to the normoxia group (Figure S2A–C). In addition, we also examined the morphological changes in BMDM in osteoclast formation under short-term hypoxia treatment (24 h exposure to 1% O_2_). Similar to RAW264.7 cells, there was a significant increase in the number of TRAP-positive multinuclear cells in the short-term hypoxia group, especially on Day 5, with the most pronounced increase and difference, compared to the normoxia group (Figure S3A–C). Thus, these results showed that either sustained or short-term hypoxic exposure could induce a significant increase in osteoclast differentiation, highlighting the sensitivity of RAW264.7 cells to hypoxic environments for osteoclastogenesis.

### 2.2. Hypoxia Dramatically Suppresses USP18 Expression in Osteoclast Differentiation

**Figure 2:**
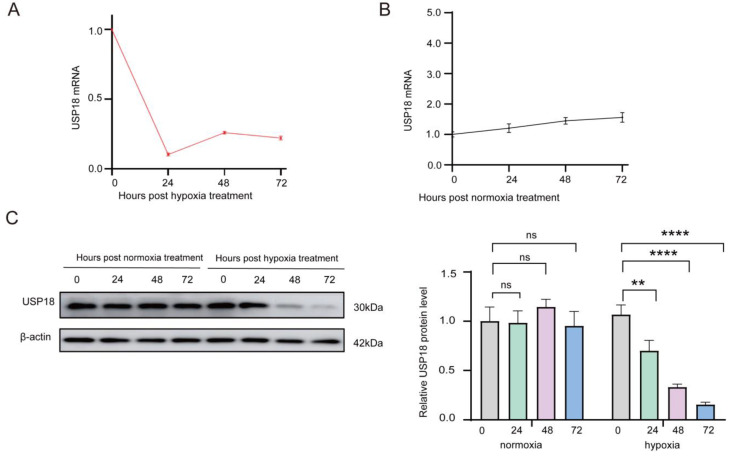
Hypoxia dramatically suppresses the USP18 expression during osteoclast differentiation. RAW264.7 cells were treated with 75 ng/mL RANKL for 0 h, 24 h, 48 h, and 72 h under normoxic (21% O_2_) or hypoxic (1% O_2_) conditions. (**A**,**B**) A qRT-PCR analysis was performed for USP18 expression. (**C**) The protein level of USP18 was detected using Western blot. Data are presented as mean ± SD (*n* = 3) (** *p* < 0.01, **** *p* < 0.0001, ns: not significant).

We also found that both USP18 mRNA and protein levels were obviously downregulated over time in response to sustained hypoxia (1% O_2_) during the differentiation of RAW264.7 cells into osteoclasts (Figure 2A,C). At the 24th hour of hypoxic stimulation, the USP18 mRNA was significantly decreased and remained at a low level through the following 48 and 72 h, whereas the USP18 mRNA under normoxic condition exhibited no variations over the same time period (Figure 2A,B). Furthermore, at the 24th hour of hypoxic stimulation, the USP18 protein was remarkably reduced and continued to be downregulated at 48 and 72 h (Figure 2C). The relative fold change in the USP18 protein at 24 h dropped from 0.90 at 0 h to approximately 0.70 at 24 h and further decreased to about 0.40 at 72 h. In contrast, under normoxia conditions, the USP18 protein level showed no changes over time (Figure 2C). Given the pivotal role of DUBs in numerous intracellular events due to its regulation of the availability and activity of functional proteins [42,43], we hypothesized that the hypoxia-induced downregulation of USP18 might be involved in hypoxia-mediated promotion in osteoclastogenesis.

Additionally, we conducted short-term hypoxic treatment (0, 6, 12, and 24 h) on the second day for osteoclast differentiation derived from RAW264.7 (Figure S4A,C) and BMDM (Figure S4B) cells. Short-term hypoxic exposure also resulted in a notable decrease in USP18 mRNA and protein levels in osteoclast differentiation from both RAW264.7 and BMDM cells. During the differentiation of RAW264.7 cells into osteoclasts, both the mRNA and protein levels of USP18 were significantly reduced at the sixth hour of hypoxic stimulation (1% O_2_) and remained at low levels for up to 24 h (Figure S4A,C). The relative fold change in the USP18 mRNA was reduced from 1.0 (0 h) to 0.1 at 6 h and further to 0.09 at 24 h (Figure S4A). Moreover, a grayscale analysis showed that the relative fold change in the USP18 protein decreased from 1.34 (0 h) to 1.13 at 6 h and to 0.70 at 24 h (Figure S4C). These results indicated that hypoxia could significantly downregulate USP18 expression during osteoclastogenesis, highlighting the potential importance of USP 18 in osteoclast differentiation under hypoxic conditions.

### 2.3. USP18 Negatively Modulates Osteoclast Differentiation

**Figure 3:**
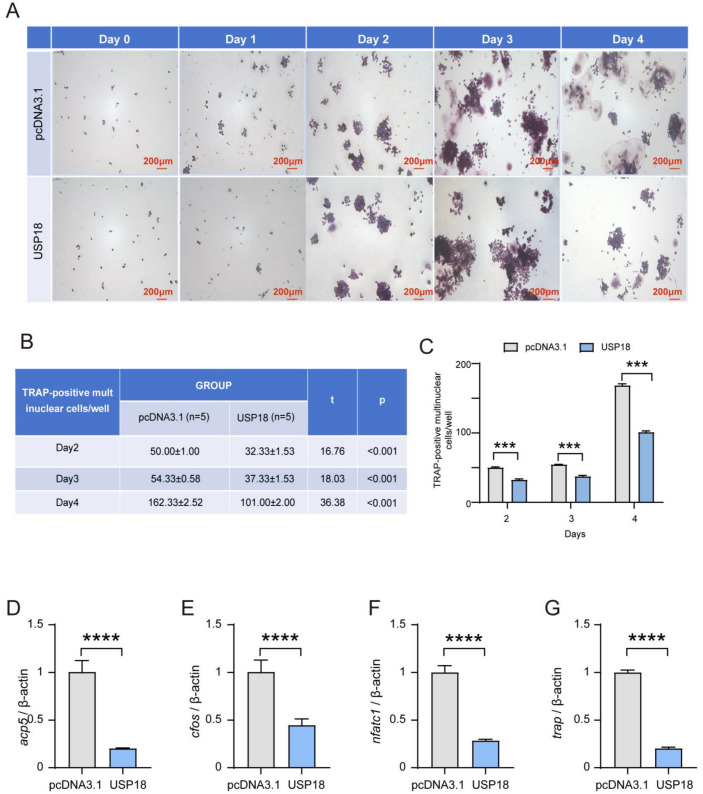
Overexpression of USP18 obviously inhibits RANKL-induced osteoclast differentiation. RAW264.7 cells were transfected with USP18 plasmid for overexpression, followed by treatment with 75 ng/mL RANKL for 0–4 days. (**A**–**C**) Osteoclastogenesis was measured using TRAP staining. Scale bar, 200 μm. *n* = 5. (**D**–**G**) qRT-PCR detected the mRNA expression of osteoclast markers using RNA isolated from RAW264.7 cells cultured in the presence of 75 ng/mL RANKL for 2 days (*n* = 3). Data are presented as mean ± SD (*** *p* < 0.001, **** *p* < 0.0001).

To gain insight into the function of USP18 in osteoclast differentiation, we transiently transfected the Usp18 plasmid to achieve its overexpression in RAW264.7 cells (Figure S5). Overexpressing USP18 notably inhibited osteoclast differentiation through TRAP staining across the observed days, with a clear reduction in TRAP-positive multinuclear cells and quantified counts (Figure 3A–C). Consistently, the overexpression of USP18 significantly downregulated the expression of osteoclast marker genes Acp5, cFos, Nfatc1, and Trap, further supporting its role in inhibiting osteoclastogenesis (Figure 3D–G). These results concluded that the overexpression of USP18 leads to a marked decrease in osteoclast differentiation and related marker gene expression, indicating its critical role in suppressing osteoclastogenesis.

**Figure 4:**
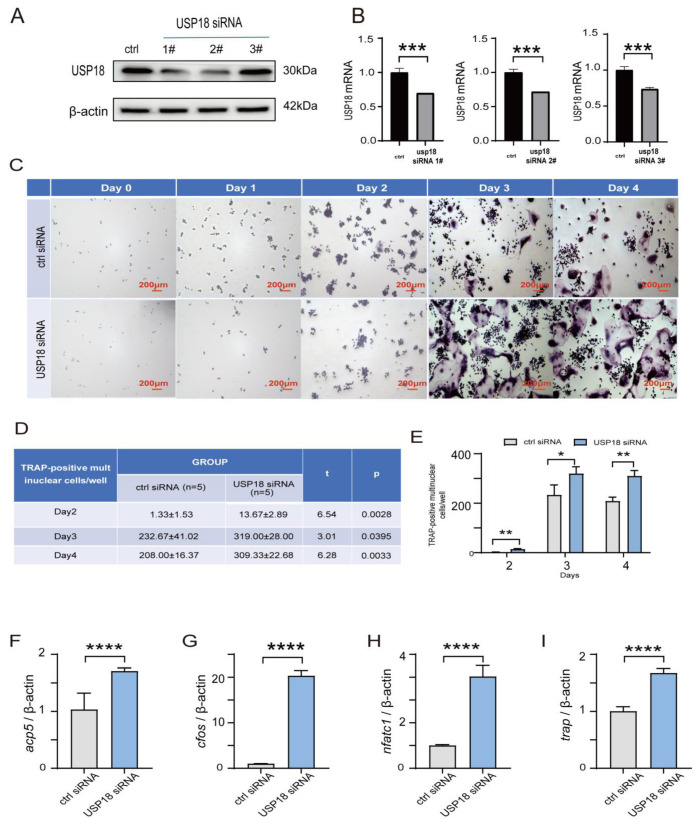
Knockdown of USP18 remarkably promotes RANKL-induced osteoclast differentiation. RAW264.7 cells were transfected with siRNA for silencing USP18, followed by treatment with 75 ng/mL RANKL for 0–4 days. (**A**) Whole-cell lysates were immunoblotted with the indicated antibodies (*n* = 3). (**B**) qRT-PCR analysis was performed for USP18 expression (*n* = 3). (**C**–**E**) Osteoclastogenesis was measured using TRAP staining. Scale bar, 200 μm. *n* = 5. (**F**–**I**) qRT-PCR detected the mRNA expression of osteoclast markers using RNA isolated from RAW264.7 cells cultured in the presence of RANKL for 2 days (*n* = 3). Data are presented as mean ± SD (* *p* < 0.05, ** *p* < 0.01, *** *p* < 0.001, **** *p* < 0.0001).

To further confirm the role of USP18 in osteoclastogenesis, we performed siRNA-mediated knockdown of USP18 in RAW264.7 cells. After transfecting the RAW264.7 cells with three siRNAs targeting USP18, we verified the knockdown efficiency by measuring the protein and mRNA levels with immunoblot and qRT-PCR, respectively (Figure 4A,B). We found that the USP18 mRNA level was downregulated after interference with all three siRNAs, whereas only the siUsp18-2 significantly reduced the protein level of USP18, prompting its selection for the subsequent experiments (Figure 4A,B). TRAP staining showed that compared to the control, the knockdown of USP18 remarkably enhanced the osteoclast differentiation across the observed days, with a significant increase in TRAP-positive multinuclear cells and quantified counts (Figure 4C–E). Consistently, RT-qPCR analysis revealed that silencing USP18 obviously facilitated the expression of osteoclast marker genes Acp5, cFos, Nfatc1, and Trap (Figure 4F–I). Our findings implicated that USP18 was an inhibitor for osteoclastogenesis, which was consistent with the abovementioned overexpression experiments.

### 2.4. Overexpressing USP18 Rescues Hypoxia-Enhanced Osteoclast Differentiation

**Figure 5:**
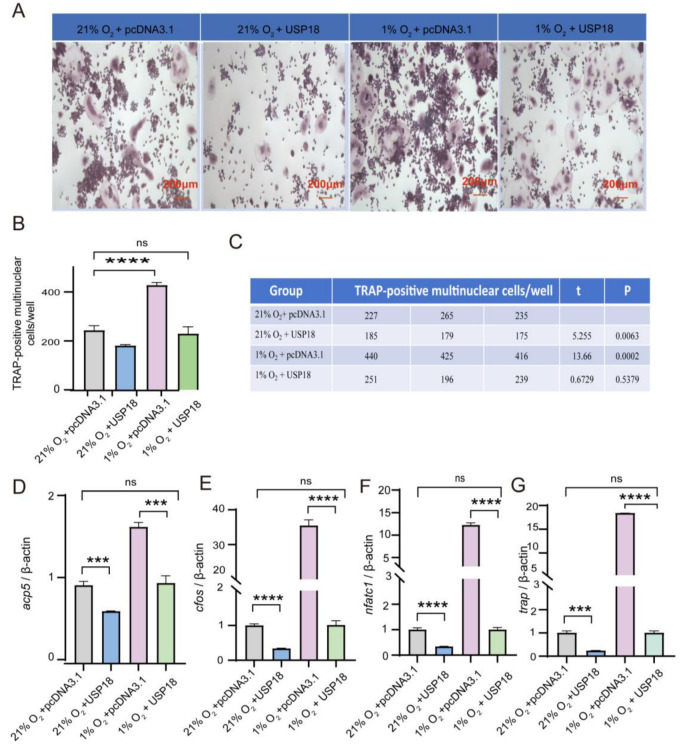
USP18 rescues hypoxia-induced promotion of osteoclast differentiation. RAW264.7 cells were transfected with USP18 plasmid for overexpression and then treated with RANKL for 2 (qRT-PCR) or 3 (TRAP staining) days under normoxic (21% O_2_) or hypoxic (1% O_2_) conditions. (**A**–**C**) Osteoclastogenesis was measured using TRAP staining. Scale bar, 200 μm. *n* = 5. (**D**–**G**) qRT-PCR detected the mRNA expression of osteoclast markers (*n* = 3). Data are presented as mean ± SD (*** *p* < 0.001, **** *p* < 0.0001, ns: not significant).

Given that the hypoxia facilitates osteoclastogenesis by suppressing the expression of USP18, an inhibitory factor of osteoclast differentiation, we sought to explore whether overexpressing USP18 is sufficient to rescue the pro-osteoclastogenic effect of hypoxia. We initially confirmed the protein levels of USP18 under normoxic and hypoxic conditions, as well as after USP18 overexpression, finding that compared to normoxia (21% O_2_ with overexpressing pcDNA3.1 vector), USP18 expression was indeed downregulated under hypoxia (1% O_2_ with overexpressing pcDNA3.1 vector), while the hypoxia-mediated downregulation of USP18 was almost reversed after USP18 overexpression (1% O_2_ with overexpressing USP18) (Figure S6). Subsequent experiments were conducted accordingly. As shown in Figure 1, the counts of osteoclasts reached their peak on Day 3, and the promoting effect of hypoxia on osteoclastogenesis was also most pronounced on this day. Therefore, we investigated the impact of USP18 overexpression on hypoxia-promoted osteoclastogenesis by performing TRAP staining on Day 3 and assessing the expression of osteoclast markers on Day 2. Under hypoxic conditions, the overexpression of USP18 resulted in a significant reduction in the number of TRAP-positive cells compared to the control group (1% O_2_ with overexpressing pcDNA3.1 vector) (Figure 5A–C). Strikingly, no significant difference was observed between the hypoxia group with USP18 overexpression and the normoxia group with overexpressing pcDNA3.1 vector. These findings indicated that overexpression of USP18 significantly reversed the hypoxia-induced increase in osteoclastogenesis. Likewise, when USP18 was overexpressed under hypoxic treatment, the expression levels of osteoclast markers, including genes Acp5, cFos, Nfatc1, and Trap, were remarkably reduced compared to the control hypoxic group (Figure 5D–G). Collectively, these results revealed that USP18 played a critical role in counteracting the effects of hypoxia on osteoclast differentiation, highlighting that the hypoxia-mediated enhancement of osteoclastogenesis was achieved by downregulating USP18 expression.

### 2.5. USP18 Inhibits Osteoclast Differentiation by Suppressing the NF-κB Signaling Pathway

**Figure 6:**
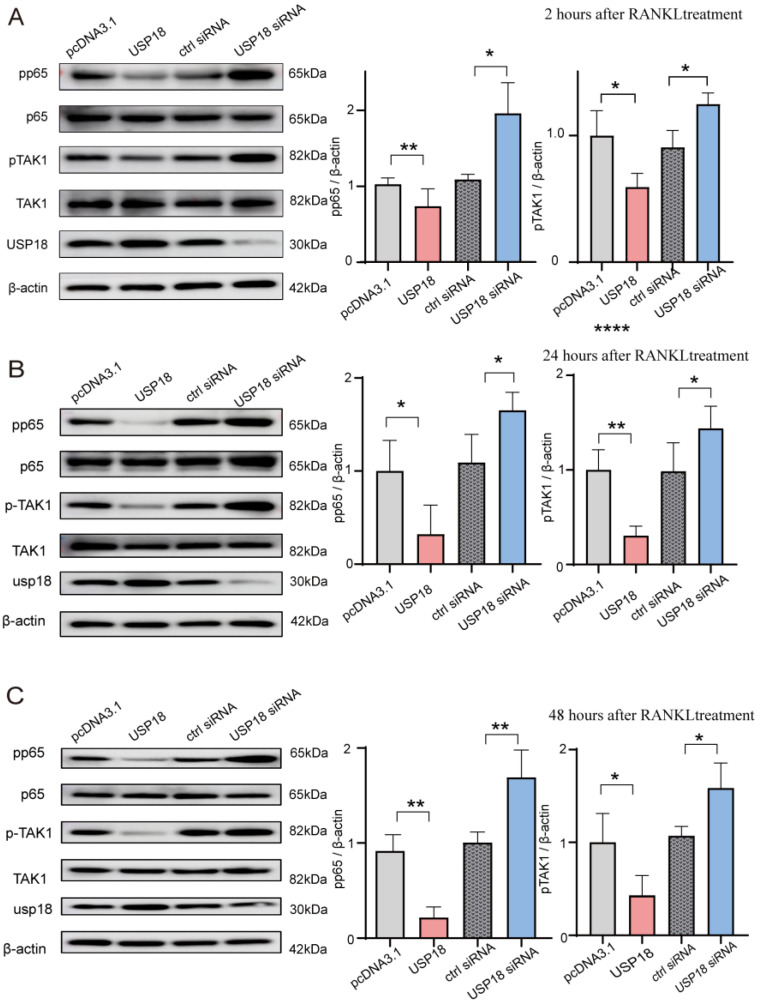
USP18 inhibits osteoclast differentiation by suppressing the NF-κB signaling pathway. (**A**–**C**) RAW264.7 cells were transfected with USP18 plasmid and siRNA for overexpression and silencing, respectively, followed by treatment with 75 ng/mL RANKL for 2 (**A**), 24 (**B**), and 48 (**C**) hours. Whole-cell lysates were immunoblotted with the indicated antibodies. Data are presented as mean ± SD, (*n* = 3) (* *p* < 0.05, ** *p* < 0.01, **** *p* < 0.0001).

The NF-κB pathway plays a crucial role in osteoclast differentiation [44]. To determine whether USP18-mediated inhibition on osteoclast differentiation is NF-κB pathway-dependent, we transfected RAW264.7 cells with Usp18 plasmid or siRNA-Usp18. Immunoblot analysis showed that overexpressing USP18 resulted in reduced activation of the NF-κB pathway, as evidenced by decreased pp65 levels following 2/24/48 h of RANKL treatment (Figure 6A–C). Conversely, increased pp65 levels were observed in the USP18 knockdown group after 2/24/48 h of RANKL treatment, leading to augmented activation of the NF-κB pathway (Figure 6A–C). These findings indicated that the USP18 protein level was inversely correlated with the activation of the NF-κB pathway, suggesting that the USP18 acts as a negative regulator of the NF-κB pathway. Additionally, pTAK1 also showed a negative correlation with the USP18 protein level (Figure 6A–C), revealing that the USP18 might target TAK1 or its upstream molecules, such as MyD88, TRIF, IRAK1, TRAF2, TRAF6, to inhibit the activation of the NF-κB pathway. In conclusion, the inhibitor effect of USP18 on osteoclast differentiation was dependent on the NF-κB signaling pathway, potentially by targeting TAK1 or its upstream adapters.

## 3. Discussion

Our study revealed that both sustained and transient hypoxia significantly promote osteoclast differentiation and contribute to the pathogenesis of osteoporosis. However, the specific impact of hypoxia on osteoclast differentiation has been inconsistent across studies, with some reporting enhanced osteoclast differentiation and activity and others showing reduced osteoclastogenesis. Prior research has indicated that hypoxia could promote the differentiation of BMDMs in osteoclast, with the peak effect observed at 2% O_2_, resulting in a fourfold increase in the number of mature multinucleated cells and a 21-fold increase in resorptive activity [14,45]. Similar results were found for the effect of hypoxia on cat osteoclast activity [45]. Furthermore, Muzylak et al. observed that despite a reduction in osteoclast numbers, bone resorption was augmented due to the enlargement of osteoclasts induced by hypoxia [46]. Conversely, Knowles et al. found that hypoxia (1% O_2_) reduced the formation and activity of osteoclasts in vitro [47]. Gorissen et al. further reported that hypoxia inhibited cellular multinucleation during osteoclast differentiation [48]. These discrepancies might be attributed to variations in the methods used to identify osteoclasts, the oxygen concentrations applied, and the duration of hypoxic treatment. Nonetheless, these findings from our group and others suggested that hypoxia may exert complex effects on osteoclast differentiation and bone resorption, which warrant further in vivo investigation.

Meanwhile, during RANKL-induced osteoclast differentiation in RAW264.7 cells, USP18 expression remained unaffected under normoxic conditions, whereas it was downregulated under hypoxic conditions. Previously, Yim et al. demonstrated that USP18 expression was upregulated after treatment with RANKL in mouse BMDMs [29]. Considering that both M-CSF and RANKL are required to induce osteoclast differentiation in BMDMs, it is challenging to determine whether the upregulation of USP18 expression is RANKL-dependent. Therefore, it is necessary to explore the underlying mechanisms of USP18 upregulation in BMDM. In addition, the role of USP18 has been reported in USP18-knockout mice, with an elevated response to type I IFN-enhancing RANKL-mediated osteoclastogenesis [29]. We propose that downregulated USP18 expression may be involved in hypoxia-induced augmented osteoclastogenesis. Further experiments confirmed that USP18 acts as a negative regulator of osteoclast differentiation, implicating that the hypoxia-mediated enhancement of osteoclast differentiation is achieved through downregulating USP18 expression. However, the precise mechanisms underlying the USP18-mediated suppression of osteoclastogenesis require further elucidation.

In addition, the inhibitory role of USP18 on osteoclastogenesis has been reported via suppressing type I IFN [29]. Originally identified as a type I interferon response gene, USP18 is rapidly upregulated via the JAK/STAT kinase pathway following IFN-β stimulation in osteoclast differentiation [49]. Moreover, USP18 acts as an inhibitor of the type I IFN signaling pathway in both ISG15-dependent [50] and -independent manners [51,52]. In addition, USP18 also acts as a novel negative regulator in toll-like receptor (TLR)-mediated NF-κB signaling by attenuating the ubiquitination of the TAK1/TAB1 complex and IKKα/β-NEMO complex in both isopeptidase-dependent and -independent ways [53]. In mammals, the NF-κB/Rel family comprises five members: NF-kB1 (p50), NF-kB2 (p52), RelA (p65), Rel-B, and c-Rel, which form homo- or heterodimers and remain as an inactive complex with IκB inhibitory proteins to block their nuclear import [54]. The most abundant form of NF-κB activated by the canonical pathway is the p65:p50 heterodimer [55]. Considering the pivotal role of the NF-κB signaling pathway in the development of various endocrine system disorders, particularly osteoporosis [56,57,58], we conducted immunoblotting to detect the activation/phosphorylation of p65 under varied USP18 expression conditions. Our findings further revealed that the USP18 serves as a negative regulator of the NF-κB signaling pathway, potentially by targeting TAK1 or its upstream adapters. The exact molecular targets of USP18 in NF-κB-related osteoclastogenesis warrant further exploration.

## 4. Materials and Methods

### 4.1. Cell Culture

RAW264.7 cells were cultured in Dulbecco’s modified eagle medium (DMEM) (Gibco, Grand Island, NY, USA, cat. C11995500BT) supplemented with 10% fetal bovine serum (FBS) (Gibco, cat. 10099141C) and 0.1 mg/mL of penicillin-streptomycin (Gibco) at 37 °C in 5% CO_2_ (Thermo Fisher Scientific, Waltham, MA, USA). Murine bone marrow-derived monocytes (BMDM) flushed from the long bones of C57BL/6 mice aged 6–8 weeks were maintained in α-minimal essential medium (α-MEM, Gibco, cat. C12571500BT) containing 10% heat-inactivated FBS, penicillin (50 U/mL), and streptomycin sulfate (50 µg/mL). BMDM were cultured as follows: First, marrow cells flushed from the long bones of mice were cultured in α-MEM containing 10% FBS in the presence of M-CSF (10 ng/mL) at 37 °C in 5% CO_2_ for 3 days. Non-adherent cells were removed by washing the culture dishes with phosphate-buffered saline, followed by incubation in the presence of M-CSF (10 ng/mL) and 75 ng/mL RANKL (RD, cat. 462-TEC-010) for 7 days for osteoclast detection. Cells were incubated in a hypoxic chamber (HERACELL 150i, Thermo Fisher Scientific, Waltham, MA, USA) at 5% CO_2_ and 1% O_2_, balanced with N_2_ as indicated or in a normal incubator containing 5% CO_2_ and approximately 20% O_2_ (Forma Series II, Thermo Fisher Scientific). Hypoxic conditions were achieved in a hypoxia chamber (memmert) flushed with a pre-analyzed gas mixture of 1% O_2_, 5% CO_2_, and 94% N_2_ or 2% O_2_, 5% CO_2_, and 93% N_2_. For the induction of osteoclast differentiation, RAW264.7 macrophages were seeded at a density of 1 × 10^4^/well in 24-well plates and cultured overnight in complete DMEM. The cells were then replaced with α-MEM/DMEM containing 75 ng/mL RANKL (RD, cat. 462-TEC-010), 10% FBS, and 0.1 mg/mL penicillin-streptomycin. The time point of the RANKL addition was designated as Day 0, and the medium was changed every other day. The corresponding days were induced according to the requirements of subsequent experiments.

### 4.2. Quantitative Real-Time Polymerase Chain Reaction (qRT-PCR)

Total RNA was isolated from preosteoclasts using TRIzol reagent (Invitrogen, cat. 15596018) and subjected to cDNA synthesis using HiScript II Q RT SuperMix for quantitative polymerase chain reaction (+gDNA wiper; Vazyme, Nanjing, China, cat. R223). qRT-PCR was performed on QuantStudio 5 flex (Thermo Fisher, Waltham, MA, USA) using 2×RealStar green power mixture (GenStar, San Francisco, CA, USA, cat. A311). Relative quantification calculations were performed using the 2^−ΔCT^ method. The gene-specific primer sets (all for murine genes) were as follows:

*cFos*, 5′-cgggtttcaacgccgacta-3′
5′-ttggcactagagacggacaga-3′;
*Acp5*, 5′-cactcccaccctgagatttgt-3′
5′-catcgtctgcacggttctg-3′;
*Nfatc1*, 5′-cagtgtgaccgaagatacctgg-3′
5′-tcgagacttgatagggacccc-3′;
*Trap,* 5′-tggtccaggagcttaactgc-3′
5′-gtcaggagtgggagccatatg-3′;
*Usp18*, 5′-caggagtccctgatttgcgt-3′
5′-caaggcatcctccagggttt-3′;
*β-actin*, 5′-tctgctggaaggtggacagt-3′
5′-cctctatgccaacacagtgc-3′.

### 4.3. Immunoblot and Antibodies

For immunoblot, whole-cell lysates were obtained using low-salt lysis buffer supplemented with protease and phosphatase inhibitors on ice, followed by centrifugation at 12,000× *g* for 5 min at 4 °C. Then the protein samples were heated at 99 °C for 10 min with SDS loading buffer (Solarbio, Beijing, China, cat. P1040) and resolved on SDS-PAGE gels. Proteins were transferred to a polyvinylidene difluoride membrane (Bio-Rad, Hercules, CA, USA, cat. 1620177). The membranes were blocked with 5% (*w*/*v*) reagent-grade nonfat milk and further incubated with the appropriate antibodies. Antibodies used in immunoblot are listed as follows: rabbit anti-USP18 (1:1000, #5348; Cell Signaling Technology, Boston, MA, USA), rabbit anti-Phospho-TAK1 (1:1000, #S412; Cell Signaling Technology), rabbit anti-TAK1 (1:1000, #D94D7; Cell Signaling Technology), rabbit anti-NF-kappa B p65 (1:1000, #D14E12; Cell Signaling Technology), rabbit anti-Phospho-NF-kappa B pp65 (1:1000, #5536; Cell Signaling Technology), and anti-β-actin mouse monoclonal antibody (1:8000, #66009; Proteintech, Wuhai, China). The immune complexes were then incubated with horseradish peroxidase-conjugated anti-mouse IgG (1:5000, #SA0001; Proteintech) or anti-rabbit IgG (1:5000, #RGAR001, Proteintech) and visualized with Immobilon reagents (Millipore, Billerica, MA, USA).

### 4.4. Tartrate-Resistant Acid Phosphatase (TRAP) Staining

After the RAW264.7 cells were induced and cultured for a certain number of days according to the experimental requirements, the medium was aspirated, and the cells were washed twice with 0.5 mL of pre-warmed PBS added along the side wall of the well. Then, 0.25 mL of pre-cooled TRAP fixative solution at 2–8 °C was added to the 24-well plate for 50 s in the dark. The cells were washed 3 times, with PBS added along the side wall of the well to remove residual fixative solution. The TRAP incubation solution was prepared by mixing AS-BI Buffer, GBC staining solution, and ACP Buffer in a ratio of 10:1:90, which was protected from light. The TRAP staining solution was added dropwise to cover the cells and incubated in a 37 °C incubator in the dark for 60 min; then, the staining solution was discarded, and the cells were washed three times with distilled water. The cells were counterstained with hematoxylin in the dark for 5 min and then washed three times with tap water and photographed under a microscope after air drying.

### 4.5. Cell Transfection

For small infected RNA, RAW264.6 cells were plated at a density of 1 × 10^5^ per well and cultured overnight at 37 °C with 5% CO_2_ for transfection. A total amount of 5 pmol siRNA per well was transfected into the cells with Lipofectamine RNAiMAX (Invitrogen, Waltham, MA, USA) according to the manufacturer’s protocol. The cells were then cultured for 24 h under normoxia conditions (supplemented with 20% O_2,_ 5% CO_2_, and 75% N_2_) before proceeding with subsequent experiments. The following target and control sequences were provided by Ribobio:

siUsp18-1 GGACGCAAAGCCTCTGAAA;
siUsp18-2 CAATCTGGAACCTGACTAA;
siUsp18-3 CCTATGGGAACCACAGATA.

For overexpression, RAW264.6 cells were plated at the same density, and 400 ng/mL plasmid was transfected into the cells with Lipofectamine2000 according to the manufacturer’s protocol for 24 h before proceeding with subsequent experiments.

### 4.6. Statistical Methods

All data were analyzed using GraphPad Prism 8.0 (GraphPad Software, La Jolla, CA, USA) and are presented as “mean ± standard deviation (SD)”. Comparisons between two groups were performed using the *t*-test, while multiple comparisons between groups were analyzed using two-way ANOVA. Statistical significance was defined as *p* < 0.05.

## 5. Conclusions

In conclusion, our study reveals a novel mechanism by which hypoxia promotes osteoclast differentiation through the downregulation of USP18, which subsequently relieves the suppression of the activation of the NF-κB pathway. The findings provide novel insights into the molecular mechanisms by which hypoxia modulated bone homeostasis and suggest potential therapeutic targets for the treatment of osteoporosis. Future studies should focus on validating these findings in vivo and exploring the potential of USP18 as a therapeutic target for the treatment of bone diseases associated with altered oxygen levels.

## Data Availability

Data are contained within the article and Supplementary Materials.

## Supplementary Materials

The following supporting information can be downloaded at https://www.mdpi.com/article/10.3390/ijms26010010/s1.
