# Supplementary material for: Hypoxia Promotes Osteoclast Differentiation by Weakening USP18-Mediated Suppression on the NF-κB Signaling Pathway
Source: Int J Mol Sci. 2024 Dec 24;26(1):10. doi: 10.3390/ijms26010010 (PMC11719700; doi:10.3390/ijms26010010)
Supplement: Supplementary file 1 [file ijms-26-00010-s001.zip › ijms-3393448-supplementary.pdf]

A

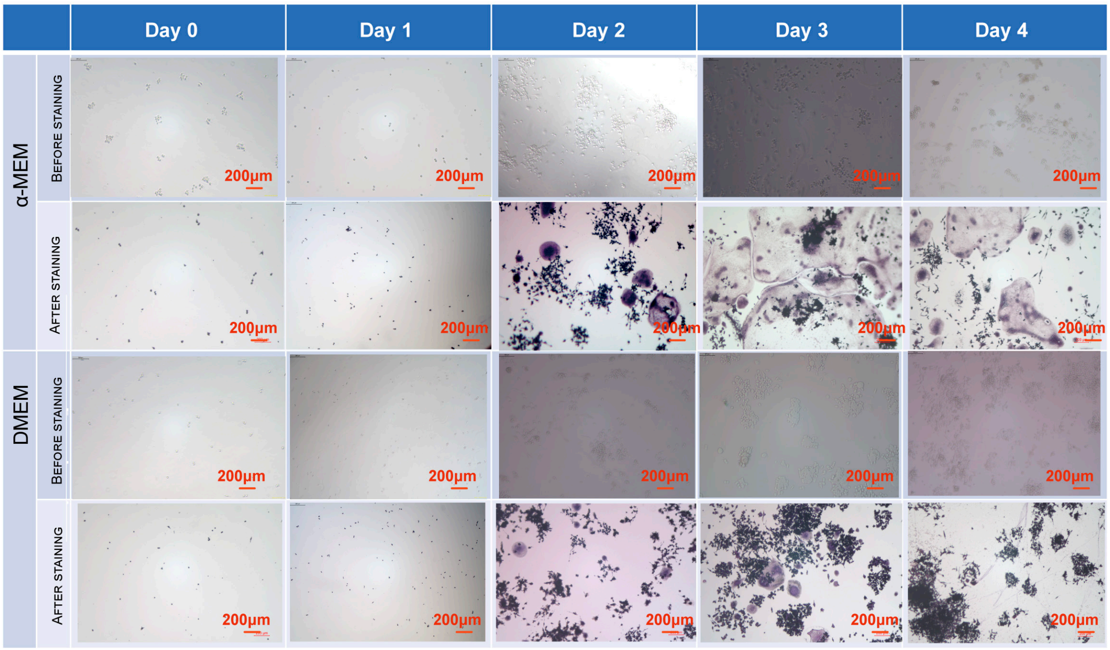

B

TRAP-positive multinuclear cells/well	GROUP		t	p
	DMEM (n=5)	α-MEM (n=5)		
Day1	0.00±0.00	1.33±0.58	4.00	0.016
Day2	2.33±0.58	61.33±12.90	7.916	0.001
Day3	9.67±4.04	128.67±12.34	17.20	<0.0001
Day4	10.67±4.93	47.33±1.53	12.30	0.0003

C

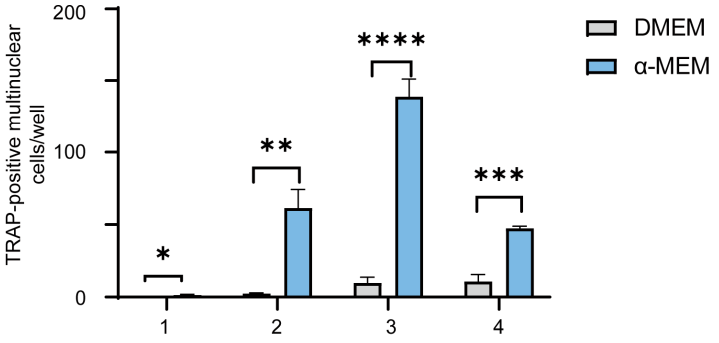

Supplementary Figure S1. Compared with the DMEM medium, the a-MEM medium is more effective in promoting RANKL-induced differentiation of RAW264.7 cells into osteoclasts. (**A**–**C**) Osteoclastogenesis was measured by TRAP

staining (Scale bar, 200 μm). Data were presented as mean ± SD (n = 5). (* $p < 0.05$, ** $p < 0.01$, *** $p < 0.001$).

A

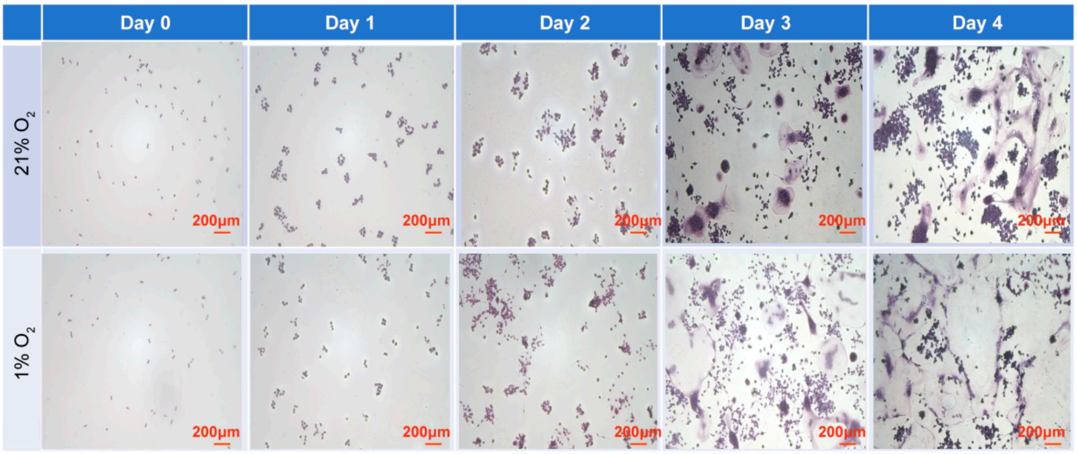

B

TRAP-positive mult inuclear cells/well	GROUP		t	p
	21%O2 (n=5)	1%O2 (n=5)		
Day2	15.00±1.00	15.50±3.50	0.24	0.8236
Day3	311.00±5.00	410.50±17.50	9.469	0.0007
Day4	162.50±25.50	219.00±1.00	3.835	0.0185

C

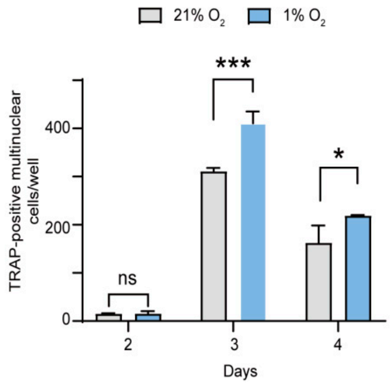

Supplementary Figure S2. Short-term hypoxia promotes osteoclastogenesis of RAW264.7. (**A**–**C**) RAW264.7 cells were treated with 75 ng/mL RANKL for 0–4 days under normoxic (21% O_2) conditions. At each time point, RANKL-induced RAW264.7 cells were placed into hypoxic incubator for 24 hours. Osteoclastogenesis was measured by TRAP staining (Scale bar, 200 μm). Data were presented as mean ± SD (n = 5). (* $p < 0.05$, *** $p < 0.001$).

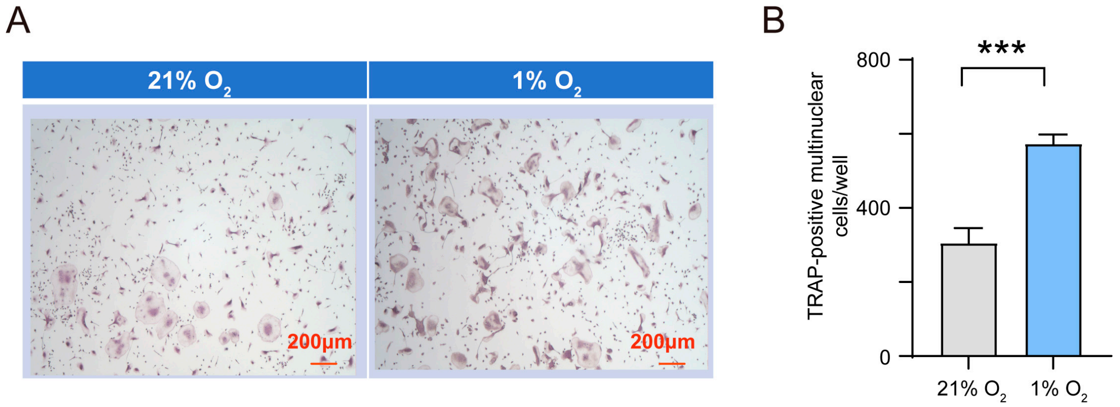

C

	21% O₂			1% O₂			t	p
D6	352	283	280	589	586	543	9.622	0.007

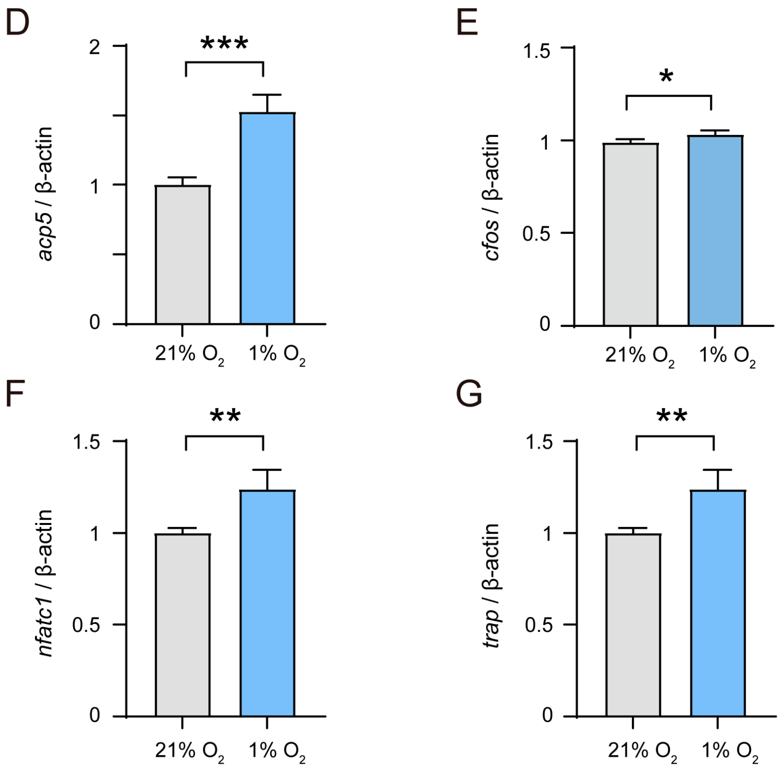

Supplementary Figure S3. Short-term hypoxia facilitates osteoclastogenesis of BMDMs. (**A**–**C**) BMDMs were cultured with 40 ng/mL M-CSF and 75 ng/mL RANKL for 5 days. Osteoclastogenesis was measured by TRAP staining (Scale bar, 200 μm; n = 5). (**D**–**G**) qRT-PCR detected the mRNA expression of osteoclast markers (n = 3). Data are presented as mean ± SD (* $p < 0.05$, ** $p < 0.01$, *** $p < 0.001$).

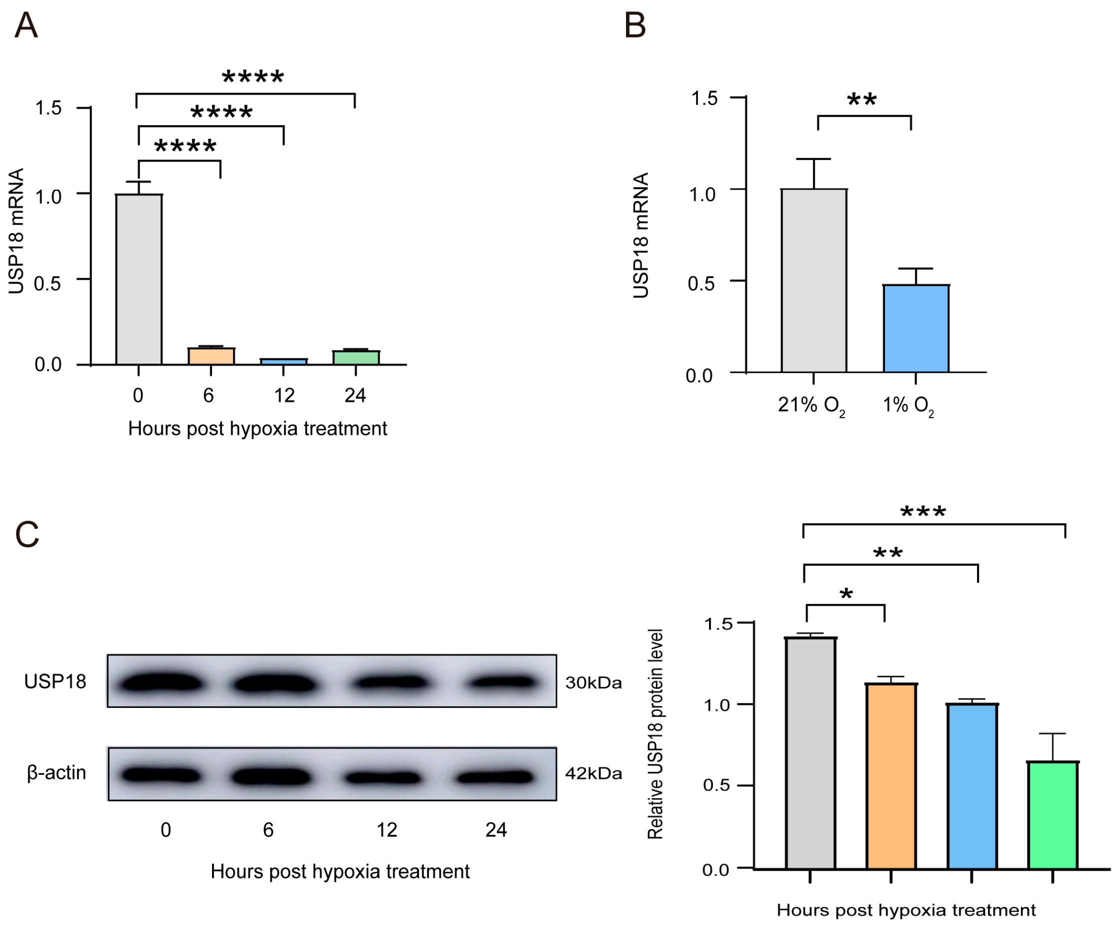

Supplementary Figure S4. Short-term hypoxia downregulates USP18 expression in osteoclast differentiation. (**A**,**B**) RANKL-induced RAW264.7 cells were treated with hypoxia (1% O_2) for 0, 6, 12, and 24 hours (**A**). BMDMs were cultured with 40 ng/mL M-CSF and 75 ng/mL RANKL for 5 days, followed by hypoxic treatment (1% O_2) for 24 hours (**B**). qRT-qPCR analysis detected the USP18 expression. (**C**) RANKL-induced RAW264.7 cells were treated with hypoxia (1% O_2) for 0, 6, 12, and 24 hours. The USP18 protein level was measured by immunoblotting with the indicated antibodies. Data were presented as mean ± SD, (n = 3). (* $p < 0.05$, ** $p < 0.01$, *** $p < 0.001$, **** $p < 0.0001$).

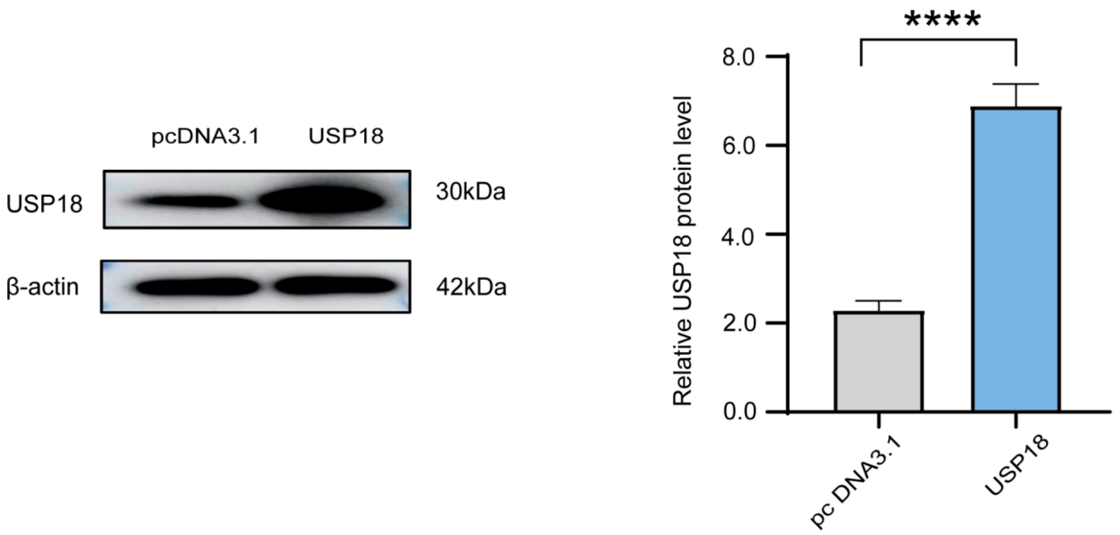

Supplementary Figure S5. Immunoblot analysis of the efficiency of USP18 overexpression in RAW264.7 cells. The protein level of USP18 was measured by immunoblotting with the indicated antibodies. Data are presented as mean ± SD, (n = 3). (**** $p < 0.0001$).

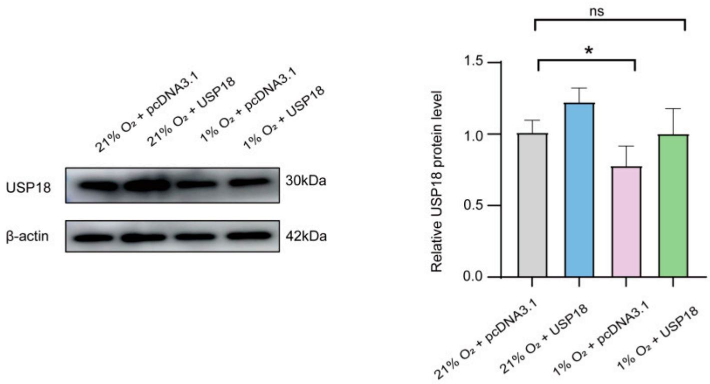

Supplementary Figure S6. Immunoblot analysis of the efficiency of USP18 overexpression in RAW264.7 cells under normoxic (21% O_2) or hypoxic (1% O_2) conditions. The protein level of USP18 was measured by immunoblotting with the indicated antibodies. Data are presented as mean ± SD, (n = 3). (* $p < 0.05$).
